# Sticky traps for *Aedes aegypti* surveillance and targeted vector control in Sincelejo, Colombia

**DOI:** 10.7705/biomedica.7290

**Published:** 2025-03-28

**Authors:** Carlos Sermeño-Correao, Alexander Bedoya-Polo, Erwin Camacho, Eduar Bejarano-Martínez

**Affiliations:** 1 Investigaciones Biomédicas, Universidad de Sucre, Sincelejo, Colombia Universidad de Sucre Colombia

**Keywords:** Aedes, Culicidae, arboviruses, entomology, mosquito vectors, public health, Aedes, Culicidae, arbovirus, entomología, mosquitos vectores, salud pública

## Abstract

**Introduction.:**

Entomological surveillance of adult *Aedes aegypti* mosquitoes provides better risk indicators than in immature stages.

**Objective.:**

To determine the usefulness of MosquiTRAP™ traps for *Ae. aegypti* surveillance, targeted vector control, and the design of dengue prevention measures in Sincelejo, Colombia.

**Materials and methods.:**

Forty-nine MosquiTRAP™ traps were deployed over six months to capture gravid *Ae. aegypti* females in two neighborhoods with historical reports of dengue cases. Entomological indices were calculated to monitor mosquito population dynamics, and the infection frequency of the captured mosquitoes with dengue, zika, and chikungunya virus were assessed. The rates of trap approval and adherence were evaluated, and risk maps were developed based on mosquito abundance. These maps facilitated the identification of specific areas for targeted vector control interventions.

**Results.:**

A total of 1,475 mosquitoes were captured, of which 99.1% were identified as *A. aegypti*. The trap positivity index ranged from 85.7 to 42.9% per inspection, with a mean female *Aedes* index of two to three mosquitoes per house. Evidence of *Ae. aegypti* infestation was observed in both neighborhoods, although specific hotspots of high mosquito abundance were identified. No viral infection was detected in the captured mosquitoes.

**Conclusions.:**

MosquiTRAP™ traps are useful for *Ae. aegypti* surveillance as a potential tool to guide vector control and prevention measures for diseases transmitted by this mosquito species.

## Resumen

**Introducción.:**

La vigilancia entomológica del estadio adulto de *Aedes aegypti* brinda mejores indicadores de riesgo que los estadios inmaduros.

**Objetivo.:**

Determinar la utilidad de las trampas MosquiTRAP™ para la vigilancia de *Ae. aegypti*, el control vectorial y la prevención del dengue en Sincelejo, Colombia.

**Materiales y métodos.:**

Durante seis meses, se instalaron 49 trampas MosquiTRAP™ para la captura de hembras grávidas de *Ae. aegypti* en dos barrios con reportes históricos de casos de dengue. Se calcularon los índices entomológicos y se estimó la frecuencia de infección para los virus del dengue, zika y chikungunya en los mosquitos capturados. Se evaluaron los porcentajes de aprobación y uso de las trampas. Se levantaron mapas de riesgo considerando la abundancia de los mosquitos y se identificaron las zonas críticas para el posible direccionamiento de intervenciones.

**Resultados.:**

Se capturaron 1.475 mosquitos, de los cuales el 99,1 % correspondió a *Ae. aegypti*. El índice de positividad de las trampas estuvo entre el 85,7 y el 42,9 %, por inspección, con un promedio de dos a tres hembras adultas por vivienda. Se evidenció una infestación de *Ae. aegypti* en ambos barrios, y se identificaron puntos críticos con alta abundancia. No se detectó infección viral en los mosquitos capturados.

**Conclusiones.:**

Las trampas MosquiTRAP™ son útiles para la vigilancia de *Ae. aegypti* como potencial herramienta para el direccionamiento de las medidas de control vectorial y la prevención de las enfermedades transmitidas por este mosquito.

Vector-borne diseases account for 17% of communicable diseases worldwide, causing over 700,000 deaths annually. Arboviruses represent a considerable public health challenge in tropical and subtropical regions. Historically, arboviruses have significantly impacted human populations, primarily through the circulation and infection of viruses from the flavivirus and alphavirus genera, such as dengue virus (DENV), yellow fever virus (YFV), West Nile virus (WNV), and the transmission of Chikungunya virus (CHIKV) in 2014, and Zika virus (ZIKV) in 2015 ^1^.

Dengue is an arboviral disease of significant concern due to its impact on morbidity, mortality, and economic burden. It is caused by dengue virus (DENV), which is transmitted predominantly in urban areas by *Aedes aegypti* and *Aedes albopictus* mosquitoes ^2^^,^^3^. In Colombia, dengue represents a public health priority because of its reemergence and intense transmission trends. The 2019 epidemics reported a higher number of cases than those observed during the 2015 outbreak ^4^. Furthermore, the presence of the vector has been documented in all departments, including at high altitudes (up to 2,302 meters above sea level) ^5^.

Dengue prevention methods are primarily focused on controlling *Ae. aegypti*. These strategies are planned and implemented by local health authorities. However, despite the implementation of surveillance and vector control programs, the number of dengue cases has continued to rise in recent years, leading to an increased burden of disease, higher economic costs, and greater pressure on the healthcare system. The significant public health threat posed by DENV requires prioritization of research and surveillance efforts. Consequently, the Pan American Health Organization (PAHO) has advocated the implementation of new tools to prevent dengue ^6^.

Traps designed to capture adult mosquitoes have emerged as effective alternatives to traditional surveillance methods, such as ovitraps and larval index surveys. The latter primarily targets the immature stages of the vector, which may be inadequate for assessing the risk of virus transmission. The MosquiTRAP™ trap (Ecovec Ltd., Belo Horizonte, Minas Gerais, Brazil) was developed in 2003 specifically for the surveillance of *Ae. aegypti* mosquitoes by exploiting the behavior of gravid females exploring oviposition sites. This trap operates without electricity and features a matte black container that exploits visual stimuli, complemented by a synthetic attractant (Atr*Aedes*) to capture female mosquitoes. Upon entering the trap and landing on the walls, the mosquitoes are ensnared on a sticky card ^7^.

MosquiTRAP™ traps have been implemented at large municipal scales, mainly in Brazil, to assess the population dynamics of *Ae. aegypti* in areas of high, medium, and low abundance ^8^^,^^9^. These traps have also been employed in marking, releasing, and recapturing studies ^10^. Multicenter studies with MosquiTRAP™ have demonstrated advantages over traditional methods, such as ovitraps and larval surveys ^11^. In Colombia, a study using MosquiTRAP™ has been conducted to examine the population dynamics of *Ae. aegypti* and investigate their natural infection with DENV ^12^.

The implementation and assessment of adult traps as innovative tools for vector surveillance would enable the measurement of their feasibility and the estimation of true risks associated with virus transmission. Additionally, these tools would enhance the targeting of control measures (such as fumigation and elimination of breeding sites), thereby improving the effectiveness of strategies and decisions related to dengue prevention, surveillance, and vector control.

The objective of this study was to evaluate the effectiveness of MosquiTRAP™ traps for *Ae. aegypti* surveillance, vector control, and the design of dengue prevention measures in Sincelejo, Colombia.

## Materials and methods

### Study area

**Figure 1:**
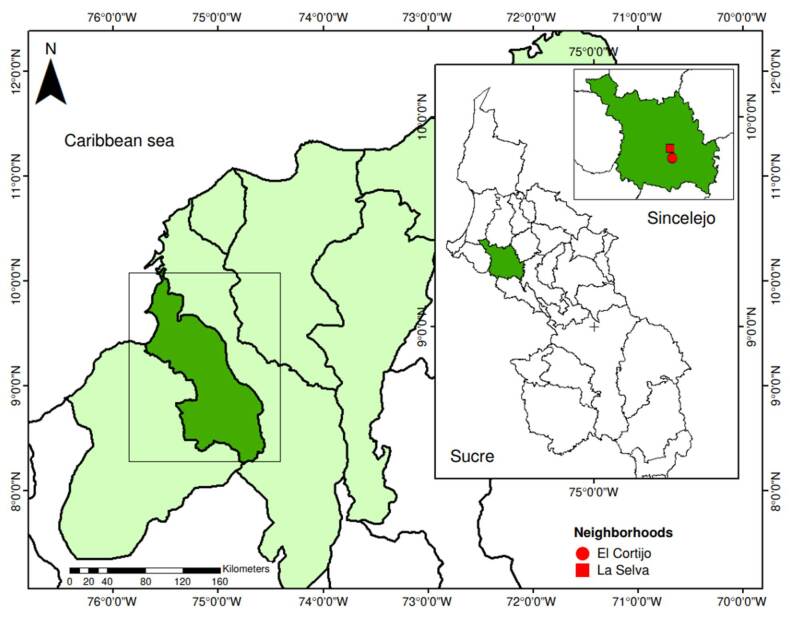
Study area and neighborhood location

This study was conducted in Sincelejo, located in the department of Sucre, in northeastern Colombia (9° 17' 58" N; 75° 23' 45" W), from May to October 2021. The municipality sits at an average altitude of 213 m.a.s.l., with an approximate annual temperature of 27°C and a relative humidity of 60% ^13^. Covering an area of 284.4 km^2^, Sincelejo has a population of 298,062 inhabitants; its urban area has nine communes and 224 neighborhoods ^13^^,^^14^. Two neighborhoods with historical reports of dengue cases were selected as mosquito collection sites: *La Selva* (9° 17’ 58.2” N; 75° 24’ 42.4” W), located at the east of the city in the commune 1, has an approximate area of 182,960 m2, an average elevation of 203 m.a.s.l. (ranging from 195 to 211 m.a.s.l.), and comprises 47 blocks with 1,084 dwellings; and *El Cortijo* (9° 16’ 59.6” N; 75° 24’ 26.9” W), located at the south of Sincelejo in the commune 3, has an approximate area of 352,462 m2, an average elevation of 201 m.a.s.l. (ranging from 189 to 211 m.a.s.l.), and consists of 63 blocks with 1,027 dwellings (figure 1).

### Installation and inspection of MosquiTRAP™ traps

For the installation of MosquiTRAP™, version 2.0, traps (Ecovec Ltd., Belo Horizonte, Minas Gerais, Brazil), the neighborhoods were initially delineated, and geospatial locations were established at equidistant points, every 100 meters, using QGIS, version 3.16.3. Each point was visited, and the closest dwellings were selected for trap installation, prior acceptance and signature the informed consent by the head of the household.

The traps were georeferenced and installed outside the houses in shaded and covered areas to protect them from the rain and sun. Each trap was filled with 300 ml of water, 10% hay infusion, and 0.08 ppm of larvicide (Dimilin™ 25 WP). After installation, the traps were inspected every 15 days over six months (from May to October 2021).

Captured mosquitoes were stored in labeled microtubes and sorted by sex and trap identification code to form pools of up to 13 individuals. The identification of the collected entomological material was initially conducted in the field and subsequently validated in a laboratory setting using the taxonomic keys proposed by Rueda ^15^ and Lane ^16^ with the aid of stereoscopic microscopes. Trap sticky cards were replaced every 30 days, and the attractant (Atr*Aedes*) was replenished every 45 days. Additionally, the water, hay infusion, and larvicide were changed during each inspection.

### Virological surveillance in collected mosquitoes

For each pool of female mosquitoes, we added 600 μl of MEM culture medium containing an antibiotic mixture (10X solution of gentamicin, penicillin, and streptomycin). The samples were then processed through mechanical maceration using the TissueLyser II™ system (QIAGEN), set at 27 rpm for 1 minute, followed by centrifugation at 12,000g for 1 minute. The supernatant from each sample was aliquoted and stored at -80°C until further use.

RNA extraction was conducted using TRIsure™ reagent (Bioline) following the manufacturer’s instructions. Briefly, 800 μl of TRIsure™ was added to each aliquot of macerate and incubated for 5 minutes at room temperature. Subsequently, 200 μl of chloroform were added, and the mixture was shaken vigorously for 15 seconds before being incubated for an additional 3 minutes at room temperature.

The samples were then centrifuged at 12,000*g* for 15 minutes at 4°C, and the aqueous phase was carefully transferred to a new vial. We added 500 μl of cold isopropanol to the aqueous phase, and incubated the mixture for 10 minutes at room temperature before centrifuging again at 12,000*g* for 10 minutes at 4°C.

The supernatant was removed, and the RNA pellet was washed with 75% ethanol. The pellet was vortexed and centrifuged at 7,500 *g* for 5 minutes at 4°C. Finally, the supernatant was discarded, and the pellet was allowed to dry at room temperature. The dry pellet was resuspended in 40 μl of ultrapure water and stored at -80°C.

RNA extracts from pools of ten mosquitoes were tested for DENV detection with real-time PCR using the Luna Universal One-Step RT-qPCR™ kit (New England Biolabs). The *Ae. aegypti* actin gene was employed as an endogenous control to ensure the integrity of the extracted RNA. Additionally, individual real-time RT-PCR assays were performed for the molecular detection of ZIKV and CHIKV.

All molecular detection assays were run on the QuantStudio 5 Real-Time PCR System™ (Thermo Fisher Scientific). The reverse transcription step was performed at 50°C for 10 minutes. The run profile consisted of an initial denaturation at 95°C for 1 minute and 40 cycles of denaturation at 95°C for 10 seconds, followed by an annealing/extension step at 58°C for 30 seconds.

The primer sequences, along with the hydrolysis probes used for the molecular detection of DENV, ZIKV, and CHIKV, and the *Ae. aegypti* actin gene, were described in previous studies ^17^^-^^20^.

### Data analysis

Entomological surveillance parameters were calculated, tabulated, and plotted by biweekly inspections and selected neighborhoods.

The parameters included:

- Trap positivity index: percentage of traps with at least one *Ae. aegypti* captured during each inspection ^21^
- Mean female *Aedes* index: average number of female *Aedes* captured per trap and per inspection ^22^
- Pending field index: percentage of MosquiTRAP™ not inspected biweekly in each neighborhood due to resident refusal or closed properties ^11^.

Risk maps were generated using QGIS, version 3.16.3. Traps with more than five captured mosquitoes in two or more inspections were identified as critical points based on the parameters established by Ritchie *et al*. ^23^ and considering a mosquito flight range of 60 m ^24^.

To assess the influence of climatic variables on mosquito abundance, daily measurements of temperature (°C), humidity (%), atmospheric pressure (mm Hg), precipitation (mm), and heat index (°C) were recorded over eight months (April to November 2021). Data was collected using a Vantage Pro2™ portable climatological station (Davis Instruments), positioned approximately 2 km from the target neighborhoods.

The data collected from the climatological variables were compared with the mean female *Aedes* index for each neighborhood, as well as overall, during various inspection periods: the week of inspection (0), two weeks before the inspection (-2), and up to four weeks before the inspection (-4). These comparisons were analyzed using the Spearman correlation, performed with the basic statistical package of R, version 4.2.1. Additionally, the approval rate for trap installation and monitoring was calculated along with adherence (the proportion of households willing to continue participating in the study).

## Results

Forty-nine MosquiTRAP™ traps were installed in the intervened neighborhoods: 21 in *La Selva* and 28 in *El Cortijo*. The approval rate for the installation and monitoring of the traps was 96%. In two households, the installation of the traps was not permitted due to security concerns and fear of contracting the coronavirus, as the study was conducted during the COVID-19 pandemic.

The adherence rate was 94%. In three dwellings, the heads of each household requested the removal of the traps due to a change of address. In all cases, the total number of traps remained constant at 49. In instances of nonapproval or withdrawal, the trap was relocated to a neighboring household.

During the six months of sampling, 12 inspections were conducted on the 49 installed traps, resulting in a pending field index of 0%, indicating that no trap was left uninspected. In total, 1,475 mosquitoes were captured in the two selected neighborhoods: 1,462 (99.1%) were identified as *Ae. aegypti*, 12 (0.8%) as *Culex* sp., and 1 (0.1%) as *Haemagogus* spp. Among the captured *Ae. aegypti*, 1,452 (99.3%) were females, and 10 (0.7%) were males. Notably, 433 (29.8%) of the collected females had fungi during trap inspection.

In terms of total female abundance per inspection in the intervened neighborhoods, a decreasing trend was observed until the tenth inspection. The highest number of mosquitoes captured occurred during the first inspection in May, with 172 mosquitoes, while the lowest count was recorded during the seventh inspection in July, with 77 mosquitoes.

**Figure 2:**
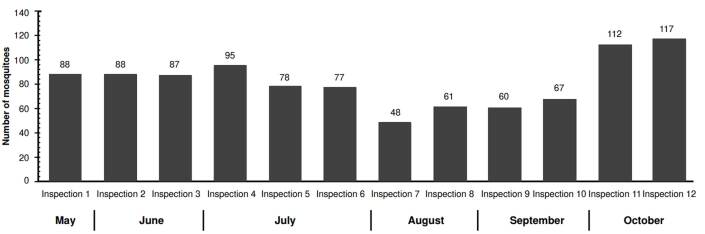
Female individuals of *Aedes aegypti* captured by inspection in *El Cortijo* neighborhood

*El Cortijo* was the neighborhood with the highest number of mosquitoes captured (978 *Ae. aegypti*) with an average of 82 mosquitoes captured per inspection. The peak number of captures occurred during the twelfth inspection, with 117 mosquitoes, while the lowest was recorded during the seventh inspection, with 48 mosquitoes (figure 2).

**Figure 3:**
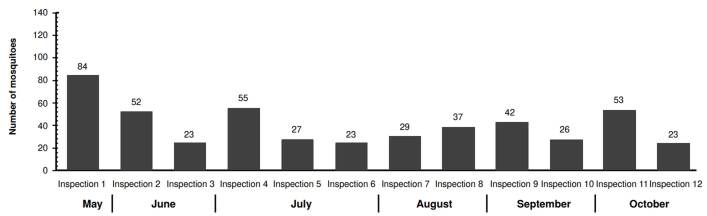
Female individuals of *Aedes aegypti* captured by inspection in *La Selva* neighborhood

**Figure 4:**
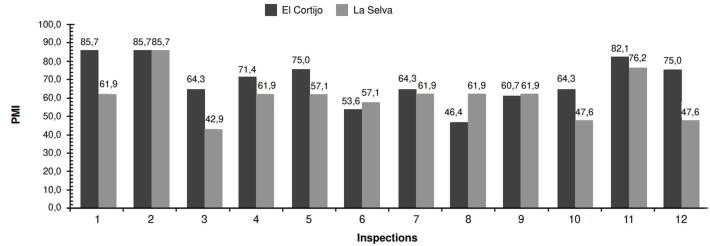
Positivity of MosquiTRAP™ traps in *La Selva* and *El Cortijo* neighborhoods

In *La Selva* neighborhood, we captured 474 mosquitoes. This population displayed more variability regarding abundance compared to *El Cortijo*. The highest capture occurred during the first inspection (84 mosquitoes), while the lowest counts (23 mosquitoes) were recorded during the third, sixth, and twelfth inspections (figure 3). The MosquiTRAP™ trap positivity -the percentage of traps capturing at least one *Ae. aegypti* mosquito- ranged from 42.9 to 85.7% across inspections, with *El Cortijo* showing a higher positivity rate (figure 4).

Nonetheless, mosquitoes were collected in 100% of the installed traps (49 out of 49) during at least one inspection in each neighborhood. The maximum number of mosquitoes captured per trap was 19 in *La Selva* during the first inspection and 43 in *El Cortijo* during the eleventh inspection.

The mean female *Aedes* index for *El Cortijo* remained constant at an average of three female mosquitoes per trap until the sixth inspection. Then, it decreased to two mosquitoes per trap during the following four inspections (7 to 10). However, it increased to four mosquitoes per trap in the eleventh and twelfth inspections.

**Figure 5:**
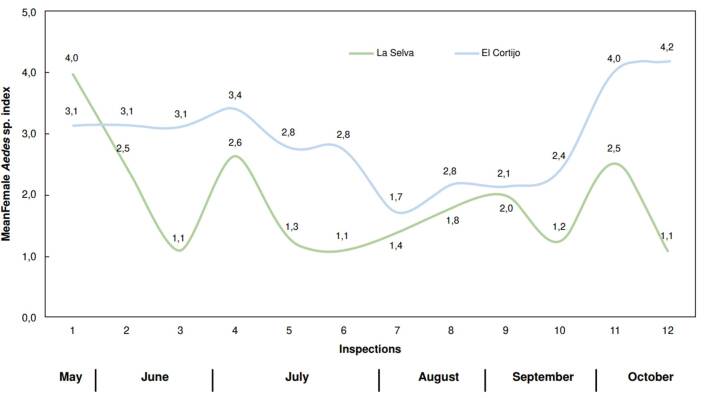
Mean female *Aedes* sp. index in *La Selva* and *El Cortijo* neighborhoods

In *La Selva* neighborhood, the mean female *Aedes* index was four female mosquitoes per trap during the first inspection. This value gradually decreased in subsequent inspections, reaching a low of one mosquito per trap by the final assessments (figure 5).

**Figure 6:**
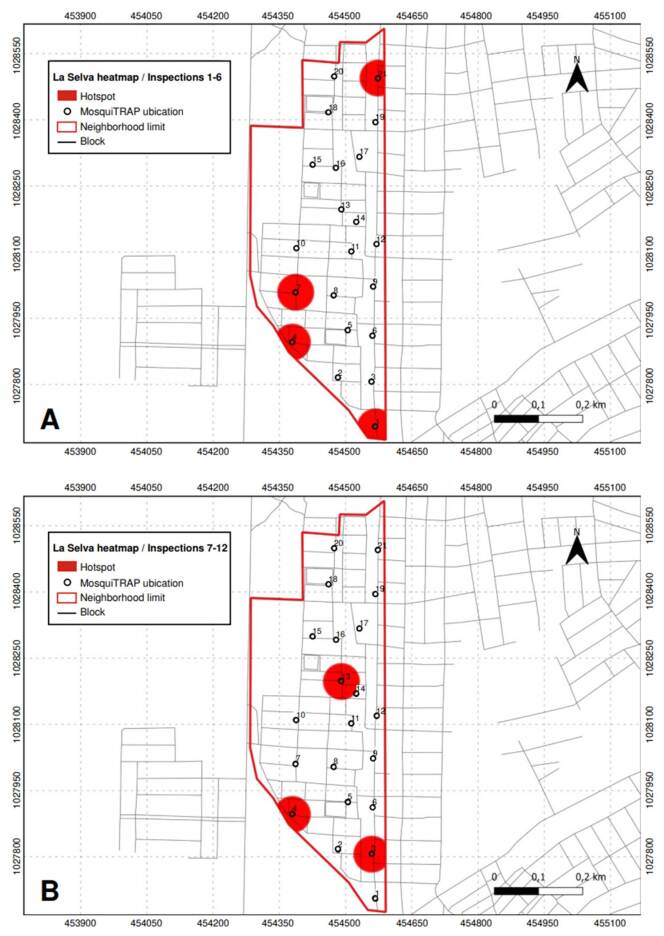
Critical points (hotspots) with high infestation of *Aedes aegypti* mosquitoes identified in *La Selva* neighborhood, Sincelejo, Colombia. A) Inspections 1 to 6; B) Inspections 7 to 12.

In *La Selva* neighborhood, four critical points were identified between the first and sixth inspections, corresponding to traps 1 (in the northern part of the neighborhood), 4, 7, and 21 (in the southern area). These traps exhibited an abundance of fewer than five mosquitoes in at least two subsequent inspections. Three additional critical points were recorded from the seventh to the twelfth inspection: two new ones at traps 3 (in the southern region) and 13 (in the center of the neighborhood). Trap 4 continued to be a critical point from earlier inspections. Five critical points were identified in *La Selva* throughout all inspections (figure 6).

**Figure 7:**
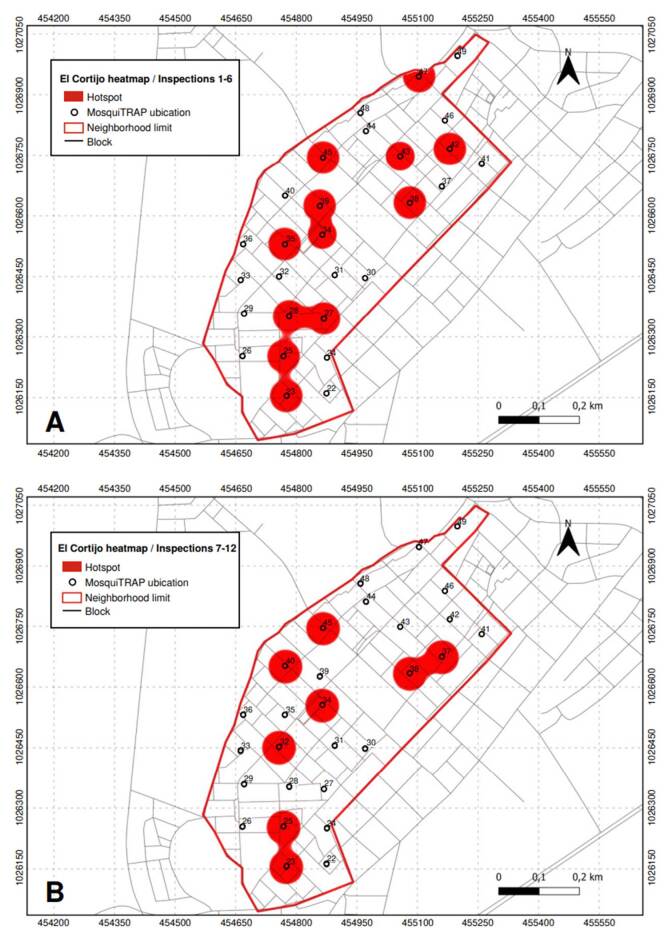
Critical points (hotspots) with high infestation of *Aedes aegypti* mosquitoes identified in *El Cortijo* neighborhood, Sincelejo, Colombia. A) Inspections 1 to 6; B) Inspections 7 to 12.

Conversely, in *El Cortijo* neighborhood, twelve critical points were identified between the first and sixth inspections, corresponding to traps 23, 25, 27, 28, 34, 35, 38, 39, 42, 43, 45, and 47. The number of critical points decreased to eight from the seventh to the twelfth inspection. Traps 23, 25, 34, 38, and 45 continued to show high infestation levels, while traps 32, 37, and 40 emerged as new critical points (figure 7).

For the molecular detection of DENV, ZIKV, and CHIKV, we processed 1,019 female mosquitoes in 110 groups. Of the analyzed mosquitoes, 731 were collected in *El Cortijo* neighborhood and 288 in *La Selva* neighborhood. The infection frequency for DENV, ZIKV, and CHIKV was zero since no viral genetic material was detected in any sample. However, amplification of the internal (*Ae. aegypti* actin gene) and positive viral controls was successfully achieved.

The analysis between climatic variables and mosquito abundance revealed a positive correlation in *La Selva*, specifically with daily precipitation (p < 0.001) and hourly precipitation (p < 0.01), measured two weeks before sampling. In contrast, no significant correlations were found for *El Cortijo* across any variable. Additionally, we determined a global negative correlation between the abundance recorded during the sampling week and the maximum atmospheric pressure (p < 0.01) (table 1).

**Table 1:** Correlation analysis between the abundance of female *Aedes aegypti* mosquitoes and climatic variables. We display data from the first week of inspection (week 0), two weeks before the inspection (week -2), and up to four weeks before the inspection (week -4). Significant correlation coefficients were highlighted in bold.

Week	Global													
	°T mean	°T max	°T min	H mean	H max	H min	AP mean	AP max	AP min	P mm	P mm/h	C mean	C max	C min
-4	0.1866	0.1789	0.2389	-0.0456	-0.0666	-0.0368	-0.1912	-0.1611	-0.2619	-0.1296	-0.1016	0.1248	0.1491	0,1378
-2	-0.1441	-0.2847	0.0618	0.2977	0.2557	0.3421	0.1862	0.2673	0.1054	0.3292	0.4623	-0.2095	-0.2877	-0,1446
0	-0.1003	-0.0385	0.0989	-0.1789	-0.2684	-0.0910	-0.5195	-0.5883*	-0.4805	-0.3187	-0.1260	0.1161	0.1821	0,2703
**Week**							***El Cortijo***							
	**°T mean**	**°T max**	**°T min**	**H mean**	**H max**	**H min**	**AP mean**	**AP max**	**AP min**	**P mm**	**P mm/h**	**C mean**	**C max**	**C min**
-4	-0.1092	-0.1719	0.0339	0.2088	0.1366	0.2509	-0.0991	0.0177	-0.2214	0.1506	0.1821	-0.0668	-0.0123	-0,0512
-2	-0.1757	-0.2250	0.0106	0.2137	0.2452	0.2842	-0.0071	0.0690	-0.1230	0.0806	0.1296	-0.0563	-0.0439	-0,1270
0	-0.0475	-0.040	0.1873	-0.0263	-0.203	0.0771	-0.3103	-0.2951	-0.3322	-0.3187	-0.0490	0.3028	0.3643	0,2085
**Week**							***La Selva***							
	**°T mean**	**°T max**	**°T min**	**H mean**	**H max**	**H min**	**AP mean**	**AP max**	**AP min**	**P mm**	**P mm/h**	**C mean**	**C max**	**C min**
-4	0.5558	0.5644	0.5735	-0.4445	-0.5071	-0.4268	-0.1957	-0.2954	-0.1413	-0.5071	-0.4718	0.4240	0.3915	0,5044
-2	-0.0883	-0.1802	-0.0888	0.2747	0.2394	0.2293	0.4920	0.5587	0.4664	0.7113**	0.6268*	-0.4496	-0.5644	-0,3901
0	-0.1381	-0.0247	-0.2131	-0.1446	0.0353	-0.1901	-0.4599	-0.5648	-0.4121	-0.1690	-0.0704	-0.2619	-0.1761	0,1137

°T mean: mean temperature; °T max: maximum temperature; °T min: minimum temperature; H mean: mean humidity; H max: maximum humidity; H min: minimum humidity; AP mean: average atmospheric pressure; AP max: maximum atmospheric pressure; AP min: minimum atmospheric pressure; P mm: precipitation (mm);

P mm/h: precipitation per hour (mm/h); C mean: average actual heat; C max: maximum actual heat; C min: minimum actual heat

* p value < 0.01

** p value < 0.001

## Discussion

This study highlights the high specificity of the MosquiTRAP™ in capturing female *Ae. aegypti* under the environmental conditions of Sincelejo. This species accounted for 99.1% of the mosquito population captured. The trap’s positivity index ranged from 42.9 to 85.7%, comparable to or even exceeding the values reported in other studies ^21^^,^^25^. The high specificity observed in Sincelejo and in studies conducted in other countries ^11^^,^^21^ confirms the utility of MosquiTRAP™ for entomological surveillance of female *Ae. aegypti*. This effectiveness is attributed to the trap’s design, which includes a matte black color that acts as a visual stimulus and an olfactory attractant (Atr*Aedes*) to mimic oviposition sites for females. Atr*Aedes*, a mixture of nonanal, decanal, and 3-methylphenol, is sealed within a tube and released steadily over approximately 45 days ^26^. Additionally, hay infusion was used in the traps as it is considered one of the most attractive media for mosquito oviposition ^7^, further increasing the likelihood of successful capture.

The effectiveness of the MosquiTRAP™ for *Ae. aegypti* surveillance has also been demonstrated against other sampling methods, such as ovitraps and manual aspirators ^26^^,^^27^. In addition, using MosquiTRAPs™ for entomological surveillance offers several advantages over traditional larval index surveys, such as reduced inspection time, which implies less labor and fewer inspectors (since each operator can inspect more houses), thereby reducing operational costs ^11^. Resende *et al*. determined an average inspection time per trap and per agent of eight minutes, while larval surveys required 24.8 minutes. In contrast, the present study reported an average inspection time of just seven minutes per trap, including travel time.

Due to operational and logistical constraints, including limited personnel during the pandemic, inspections were conducted biweekly. Although MosquiTRAP™ traps are designed for weekly inspections, some studies indicate that this frequency may not be essential for optimal results in effective mosquito surveillance ^24^^,^^28^.

Previous entomo-virological surveillance studies using MosquiTRAP™ traps successfully detected dengue virus genetic material in mosquitoes captured during weekly or biweekly surveys. In the present study, 29.8% of the collected females had fungi during the trap inspection. Similarly, in a Colombian study with biweekly inspections, 6.7% of the mosquitoes were contaminated with fungi ^12^^,^^29^. This fungal contamination could be due to prolonged exposure to varying climatic conditions, such as high humidity (typical of tropical regions) and the hay infusion in the traps, which may have promoted the decomposition and growth of microorganisms.

Regarding inspection time, very short inspection intervals may reduce the sensitivity of the traps, while extended periods can increase the detection but also the variance of the data ^21^. Weekly inspections tend to preserve the anatomical structure of mosquitoes, aiding in their identification and often capturing more live specimens. In contrast, longer inspection intervals can result in a higher proportion of dead mosquitoes, poorly preserved or contaminated with fungi, delaying their identification and potentially introducing biases into the calculated entomological indicators. Additionally, the distance between traps was halved from the manufacturer’s recommendation ^28^ to increase the likelihood of mosquito capture and provide a more detailed understanding of vector population dynamics in the study areas.

The pending field index observed in this study was lower compared to others ^11^, likely due to strong community support and the designed strategy. This strategy involved contacting the head of the household by phone if the dwelling was closed during the initial visit, allowing to reschedule it within 24 hours. Additionally, inspections were conducted during the late afternoon (15:00 to 18:00), when residents were usually more available. The spatial distribution of mosquitoes was uneven in both neighborhoods, depending on the availability of food sources, oviposition sites, and environmental conditions.

Regarding vector abundance, a higher number of mosquitoes was observed in *El Cortijo* neighborhood, where dengue cases are reported annually, according to records from the *Secretaría de Salud de Sincelejo* (unpublished data). Previous studies conducted in commune 3, where *El Cortijo* is located, revealed that 98% of the inspected households have favorable conditions for mosquito breeding, such as water storage tanks and unserviceable items containing stagnant water. Additionally, Cabarca *et al*. established that the number of breeding sites significantly correlates with the presence of larvae and adult female mosquitoes ^30^.

MosquiTRAP™ traps have proven to be useful not only for estimating *Ae. aegypti* abundance but also for detecting the circulation of arboviruses, such as DENV, ZIKV, and CHIKV, within mosquito populations ^31^^,^^32^. In this study, no viral detection of DENV, ZIKV, or CHIKV was observed in the processed mosquitoes. The absence of these arboviruses can be due to their low circulation in the study areas, which aligns with the data from the endemic channel for dengue in Sincelejo. Until epidemiological week 40 (early October), dengue cases were within the “success zone”, indicating a low number of cases. However, a case increase was observed starting in October, moving into the “alert zone” ^33^. This decline in cases earlier in the year may be linked to herd immunity acquired during the dengue epidemic in 2019.

Despite the results mentioned above, data on population dynamics coupled with viral detection is crucial for controlling the spread of arboviral diseases in critical areas. Such information acts as an early warning system for potential epidemics or outbreaks, providing real-time data to enhance response efforts ^31^. The absence of viral detection for DENV, ZIKV, or CHIKV in processed mosquitoes underscores the necessity for ongoing entomo-virological surveillance.

Although DENV was not detected in the mosquitoes, two laboratory- confirmed cases of dengue fever were reported during the fifth inspection on June 15 in *El Cortijo*. The affected individuals -a 14-year-old girl and a 16-year-old boy- resided in the same house near trap 42, identified as a hotspot since the third inspection. This fact underscores the traps’ utility for entomological surveillance and the early prediction of dengue cases, given the high likelihood of outbreaks linked to vector abundance. This correlation has also been demonstrated in Belo Horizonte (Minas Gerais, Brazil), where MosquiTRAP™ traps were utilized alongside geographic information systems for spatiotemporal cluster analysis ^34^.

Geographic information systems have become increasingly important as a public health tool over the past decade, owing to their ability to enhance our understanding of epidemiology, ecology, and risk factors associated with infectious agents. The integration of geographic information systems with vector surveillance has facilitated the creation of risk maps, which are instrumental in identifying, prioritizing, and efficiently intervening in specific locations or regions at risk ^35^.

In this study, the risk maps enabled the identification and geo-referencing of critical points with high vector abundance. This information can facilitate targeted prevention and control interventions, such as biological and chemical control, as well as educational sessions, among others. Focusing on specific areas rather than implementing blanket interventions across entire neighborhoods is a more efficient way of using resources. This targeted approach improves the effectiveness of interventions while optimizing the limited economic and human resources typically available.

Positive correlations between vector abundance and daily and hourly precipitation were observed only in *La Selva*. This association can be due to the direct relationship between high rainfall and the increased availability of mosquito breeding sites. In contrast, such a correlation was not found in *El Cortijo*, likely due to the abundance of breeding sites that do not rely on rainfall to sustain high mosquito populations ^30^. Regarding the negative correlation between maximum atmospheric pressure and vector abundance, some studies report that atmospheric pressure affects the flight responses and mortality of *Ae. aegypti*^36^^,^^37^.

MosquiTRAP™ traps have demonstrated their efficiency and specificity as a surveillance tool for *Ae. aegypti* in the evaluated urban areas. This finding is a significant advancement in vector surveillance methodologies due to their practicality, compact size, and capacity for *in situ* collection (allowing taxonomic identification). Also, they reduce inspection time and enable adult mosquito capture for viral surveillance, providing reliable risk indicators. Furthermore, the use of geographic information systems allowed the creation of maps to visualize critical points for targeted prevention and control measures.

Future studies in Colombia should optimize the use of MosquiTRAP™ traps in fieldwork, considering factors such as trap density per household, minimum required distance between traps, and inspection frequency in areas with low and high vector abundance across different geographical zones.

## References

[1] World Health Organization (2022). About vector-borne diseases.

[2] Lwande O, Obanda V, Lindstrom A, Ahlm C, Evander M, Naslund J (2020). Globe-trotting Aedes aegypti and Aedes albopictus: Risk factors for arbovirus pandemics. Vector Borne Zoonotic Dis.

[3] Ferreira-de-Lima V, Lima-Camara T (2018). Natural vertical transmission of dengue virus in Aedes aegypti and Aedes albopictus: A systematic review. Parasit Vectors.

[4] Pan American Health Organization (2021). Epidemiological update dengue.

[5] Ruiz-López F, González-Mazo A, Vélez-Mira A, Gómez GF, Zuleta L, Uribe S (2016). Presence of Aedes (Stegomyia) aegypti (Linnaeus, 1762) and its natural infection with dengue virus at unrecorded heights in Colombia. Biomédica.

[6] Pan American Health Organization (2021). Métodos de vigilancia entomológica y control de los principales vectores en las Américas.

[7] Gama R, Silva I, Resende M, Eiras A. (2007). Evaluation of the sticky MosquiTRAP™ for monitoring Aedes aegypti (Diptera: Culicidae) in the district of Itapoã, Belo Horizonte, Minas Gerais, Brazil. Neotrop Entomol.

[8] Codeço CT, Lima AW, Araújo SC, Lima JBP, Maciel-de-Freitas R, Honório NA (2015). Surveillance of Aedes aegypti: Comparison of house index with four alternative traps. PLoS Negl Trop Dis.

[9] Honório NA, Codeço CT, FdC Alves, MdA Magalhães, Lourenço-De-Oliveira R. (2009). Temporal distribution of Aedes aegypti in different districts of Rio de Janeiro, Brazil, measured by two types oftraps. J Med Entomol.

[10] Maciel-de-Freitas R, Eiras ÁE, Lourenço-de-Oliveira R. (2008). Calculating the survival rate and estimated population density of gravid Aedes aegypti (Diptera, Culicidae) in Rio de Janeiro, Brazil. Cad Saude Publica.

[11] Resende MC, Silva IM, Eiras ÁE. (2010). Avaliação da operacionalidade da armadilha MosquiTRAP™ no monitoramento de Aedes aegypti. Epidemiol Serv Saude.

[12] Manjarres M, Martínez J. (2015). Dinámica poblacional y búsqueda de infección natural con virus dengue en poblaciones de *Aedes aegypti* en el municipio de Sincelejo: dos herramientas para la estimación del riesgo epidemiológico (tesis).

[13] Aguilera-Díaz M. (2005). La economía del departamento de Sucre: ganadería y sector público.

[14] Departamento Administrativo Nacional de Estadística - DANE Proyecciones y retroproyecciones de población municipal para el periodo 1985-2017 y 2018-2035 con base en el CNPV 2018.

[15] Rueda LM. (2004). Pictorial keys for the identification of mosquitoes (Diptera: Culicidae) associated with dengue virus transmission. Zootaxa.

[16] Lane J. (1953). Neotropical Culicidae.

[17] Roehrig JT, Butrapet S, Liss NM, Bennett SL, Luy BE, Childers T (2013). Mutation of the dengue virus type 2 envelope protein heparan sulfate binding sites or the domain III lateral ridge blocks replication in Vero cells prior to membrane fusion. Virology.

[18] Lanciotti RS, Kosoy OL, Laven JJ, Velez JO, Lambert AJ, Johnson AJ (2008). Genetic and serologic properties of Zika virus associated with an epidemic, Yap State, Micronesia, 2007. Emerg Infect Dis.

[19] Waggoner JJ, Ballesteros G, Gresh L, Mohamed-Hadley A, Tellez Y, Sahoo MK (2016). Clinical evaluation of a single-reaction real-time RT-PCR for pan-dengue and chikungunya virus detection. J Clin Virol.

[20] Wu P, Sun P, Nie K, Zhu Y, Shi M, Xiao C (2019). A gut commensal bacterium promotes mosquito permissiveness to arboviruses. Cell Host Microbe.

[21] Resende MC, Ázara TM, Costa IO, Heringer LC, Andrade MR, Acebal JL (2012). Field optimisation of MosquiTRAP™ sampling for monitoring Aedes aegypti Linnaeus (Diptera: Culicidae). Mem Inst Oswaldo Cruz.

[22] Lana RM, Morais MM, Lima TFMd, Carneiro TGdS, Stolerman LM, dos Santos JP (2018). Assessment of a trap based Aedes aegypti surveillance program using mathematical modeling. PLoS One.

[23] Ritchie SA, Long S, Smith G, Pyke A, Knox TB. (2004). Entomological investigations in a focus of dengue transmission in Cairns, Queensland, Australia, by using the sticky ovitraps. J Med Entomol.

[24] Winskill P, Carvalho DO, Capurro ML, Alphey L, Donnelly CA, McKemey AR. (2015). Dispersal of engineered male Aedes aegypti mosquitoes. PLoS Negl Trop Dis.

[25] Fávaro EA, Dibo MR, Mondini A, Ferreira AC, Barbosa AA, Eiras ÁE (2006). Physiological state of Aedes (Stegomyia) aegypti mosquitoes captured with MosquiTRAPsTM in Mirassol, São Paulo, Brazil. J Vector Ecol.

[26] Quimbayo M, Rúa-Uribe G, Parra-Henao G, Torres C. (2014). Evaluation of lethal ovitraps as a strategy for Aedes aegypti control. Biomédica.

[27] Fávaro EA, Mondini A, Dibo MR, Barbosa AA, Eiras ÁE, Neto FC. (2008). Assessment of entomological indicators of Aedes aegypti (L.) from adult and egg collections in São Paulo, Brazil. J Vector Ecol.

[28] Eiras ÁE, Resende MC. (2009). Preliminary evaluation of the “Dengue-MI” technology for Aedes aegypti monitoring and control. Cad Saude Publica.

[29] Pessoa A. (2007). Monitoramento do dengue virus circulante em larvas e mosquitos adultos de *Aedes aegypti*.

[30] Cabarca S, Pérez C, Blanco-Tuirán P, Castellar A, Camacho-Burgos E. (2015). Infestación por Aedes aegypti en una localidad del municipio de Sincelejo, departamento de Sucre. Rev Investig Med Trop.

[31] Eiras AE, Resende MC, Acebal JL, Paixão KS. (2018). New cost-benefit of Brazilian technology for vector surveillance using trapping system.

[32] Dos Santos TP, Cruz OG, da Silva KAB, de Castro MG, de Brito AF, Maspero RC (2017). Dengue serotype circulation in natural populations of Aedes aegypti. Acta Tropica.

[33] Instituto Nacional de Salud Estadísticas de vigilancia rutinaria.

[34] de Melo DPO, Scherrer LR, Eiras ÁE. (2012). Dengue fever occurrence and vector detection by larval survey, ovitrap and MosquiTRAP™: A space-time clusters analysis. PLoS One.

[35] Louis VR, Phalkey R, Horstick O, Ratanawong P, Wilder-Smith A, Tozan Y (2014). Modeling tools for dengue risk mapping - A systematic review. Int J Health Geogr.

[36] Haufe W. (1954). The effects of atmospheric pressure on the flight responses of Aedes aegypti (L.). Bull Entomol Res.

[37] Galun R, Fraenkel G. (1961). The effect of low atmospheric pressure on adult Aedes aegypti and on housefly pupae. J Insect Physiol.

